# Network pharmacology and molecular docking analysis reveal insights into the molecular mechanism of shiliao decoction in the treatment of cancer-associated malnutrition

**DOI:** 10.3389/fnut.2022.985991

**Published:** 2022-08-25

**Authors:** Sidan Long, Shuangshuang Ji, Peng Xue, Hongting Xie, Yinjie Ma, Shijie Zhu

**Affiliations:** ^1^Graduate School, Beijing University of Chinese Medicine, Beijing, China; ^2^Medical Oncology, Wangjing Hospital of China Academy of Chinese Medical Sciences, Beijing, China

**Keywords:** shiliao decoction, cancer-associated malnutrition, network pharmacology, molecular docking, immunity, prognosis

## Abstract

**Purpose:**

Shiliao Decoction (SLD) was developed for treatment and prevention of cancer-associated malnutrition (CAM) in China. In this study, we aim to discover SLD’s active compounds and demonstrate the mechanisms of SLD that combat CAM through network pharmacology and molecular docking techniques.

**Methods:**

All components of SLD were retrieved from the pharmacology database of Traditional Chinese Medicine Systems Pharmacology (TCMSP). The GeneCards database and the Online Mendelian Inheritance in Man database (OMIM) were used to identify gene encoding target compounds, and Cytoscape was used to construct the drug compound–target network. The network of target protein-protein interactions (PPI) was constructed using the STRING database, while gene ontology (GO) functional terms and the Kyoto Encyclopaedia of Genes and Genomes (KEGG) pathways associated with potential targets were analyzed using a program in R language (version 4.2.0). Core genes linked with survival and the tumor microenvironment were analyzed using the Kaplan–Meier plotter and TIMER 2.0 databases, respectively. Protein expression and transcriptome expression levels of core gene were viewed using the Human Protein Atlas (HPA) and the Cancer Genome Atlas (TCGA). A component-target-pathway (C-T-P) network was created using Cytoscape, and Autodock Vina software was used to verify the molecular docking of SLD components and key targets.

**Results:**

The assembled compound–target network primarily contained 134 compounds and 147 targets of the SLD associated with JUN, TP53, MAPK3, MAPK1, MAPK14, STAT3, AKT1, HSP90AA1, FOS, and MYC, which were identified as core targets by the PPI network. KEGG pathway analysis revealed pathways involved in lipid and atherosclerosis, the PI3K/Akt signaling pathway, and immune-related pathways among others. JUN is expressed at different levels in normal and cancerous tissues, it is closely associated with the recruitment of different immune cells and has been shown to have a significant impact on prognosis. The C-T-P network suggests that the active component of SLD is capable of regulating target genes affecting these related pathways. Finally, the reliability of the core targets was evaluated using molecular docking technology.

**Conclusion:**

This study revealed insights into SLD’s active components, potential targets, and possible molecular mechanisms, thereby demonstrating a potential method for examining the scientific basis and therapeutic mechanisms of TCM formulae.

## Introduction

Many malnourished cancer patients exhibit associated pathophysiological changes such as inflammation and immunosuppression, and a complex interplay exists between these two factors and cancer-associated malnutrition (CAM). Localized tumor cells and immune cells in the microenvironment interact to release various types of cytokines that promote systemic inflammation, contributing to malnutrition and poor outcomes by causing anorexia, altering metabolism, and increasing resting energy expenditure and muscle breakdown (1). According to the results of the largest global survey to date (2), the overall prevalence of malnutrition among inpatients with cancer in China is 80.4%, and the prevalence of moderate and severe malnutrition is as high as 58.2% (3). Malnutrition, as the most common clinically neglected comorbidity in tumor patients, is a major prognostic predictor of poor clinical outcomes for cancer patients.

Nutrition and health have been incorporated into China’s national strategy in “Health China 2030” and the “National Nutrition Plan (2017-2030),” which place particular focus on rapidly improving the nutrition of cancer patients. Although there is a great need for effective therapeutic options to address CAM, there is a lack of pharmacological agents that can be safely administered as part of long-term treatment. Traditional Chinese medicine (TCM) is a major component of complementary alternative medicine and can play important roles in the entire cancer treatment process, up to and including palliative care in advanced stages. Our team has long been concerned about the metabolic and immune disturbances in tumor patients and have previously designed a therapy formula named Shiliao Decoction (SLD) aimed at improving nutrition levels, drawing on the ideas behind the Wu Zhi An Zhong and Da Jian Zhong decoctions (4). Previous single-armed clinical studies have shown that SLD has outstanding efficacy in improving patients’ appetite and relieving pain, as well as synergistic effects with anti-tumor therapy.

**FIGURE 1:**
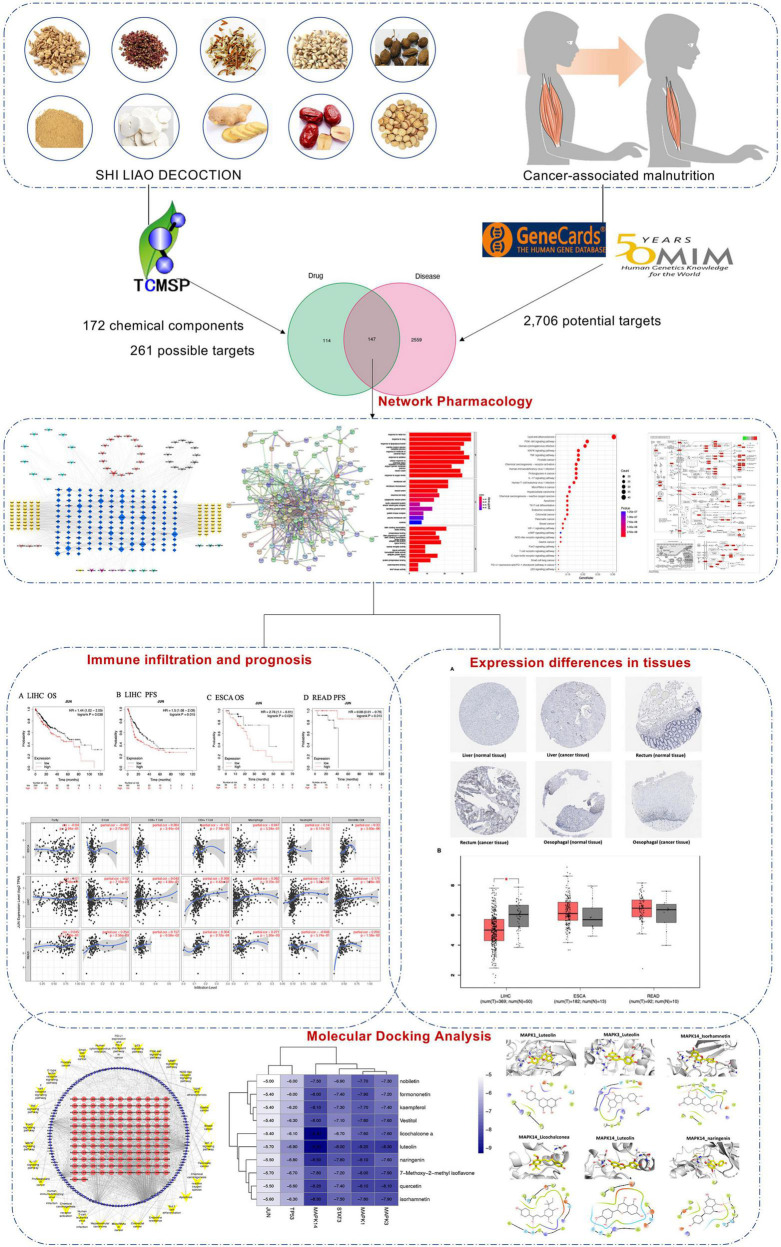
Workflow diagram of network pharmacology analysis.

Network pharmacology can create a relationship prediction model between drugs and disease targets, integrate an interaction network to analyze drug interactions with specific nodes in each network module, and investigate the interaction relationship between drugs and potential targets from a systematic perspective (5, 6). Network pharmacology is highly suited for evaluating the numerous components, targets, and pathways of TCM due to its complex composition and multitarget therapeutic characteristics (7, 8). Computer simulation technology is used in molecular docking (9). In this study, we sought to use network pharmacology to understand the active compounds of SLD and predict their potential targets and signaling pathways for the treatment of CAM. Molecular docking analysis techniques were used to validate previously obtained targets. In addition, the structural docking of related proteins and compounds has the potential to provide a theoretical basis for the development of new drugs containing the active alkaloids of botanical drugs (Figure 1).

## Materials and methods

### Composition of shiliao decoction

SLD is a decoction of 10 botanicals extracted by water, including *Codonopsis pilosula* (Franch.) Nannf (Campanulaceae; Codonopsis radix), *Zanthoxylum bungeanum* Maxim (Rutaceae; Zanthoxylum bungeanum pericarp), *Citrus reticulata* Blanco (Rutaceae;Citri reticulatae pericarpium), *Coix lacryma-jobi L.var.ma-yuen* (Roman.) Stapf (Gramineae; Coicis semen), *Dioscorea opposite* Thunb (Dioscoreaceae; Dioscoreae rhizoma), *Typha angustifolia* L.(Typhaceae; Typhae pollen), *Amomum villosum* Lour (Zingiberaceae; Amomi fructus), *Zingiber officinale* Rosc (Zingiberaceae; Zingiberis rhizome recens), *Ziziphus jujuba* Mill. (Rhamnaceae; Jujubae fructus), *Glycyrrhiza uralensis* Fisch (Leguminosae; Glycyrrhiza radix et rhizoma), all of which are plants known to have medicinal properties. Details on the composition of SLD can be found in Table 1. The plant taxonomy was confirmed using the MPNS database^1^ and drug names were confirmed using the *Chinese Pharmacopeia 2020 edition.*

**TABLE 1:** The composition of SLD.

Species	Drug name	Part used	Dosage (g)
*Codonopsis pilosula* (Franch.) Nannf.	Dangshen	Root	20
*Zanthoxylum bungeanum* Maxim.	Huajiao	Pericarp	10
*Citrus reticulata* Blanco.	Chenpi	Pericarp	10
*Coix lacryma-jobi L.var.ma-yuen* (Roman.) Stapf.	Yiyiren	Semen	30
*Dioscorea opposite* Thunb.	Shanyao	Root	15
*Typha angustifolia* L.	Puhuang	Pollen	10
*Amomum villosum* Lour.	Sharen	Fruit	10
*Zingiber officinale* Rosc.	Shengjiang	Root	5
*Ziziphus jujuba* Mill.	Dazao	Fruit	10
*Glycyrrhiza uralensis* Fisch.	Gancao	Root	10
Total			130

### Database establishment

Active components of SLD were identified in the Traditional Chinese Medicine Systems Pharmacology (TCMSP) Database and Analysis Platform (TCMSP)^2^ (10). Oral bioavailability (OB) refers to the rate and extent at which a drug is absorbed into the body’s circulatory system. Drug-like (DL) properties reflect the nature of a drug that has a specific functional group or contains the same or similar physical characteristics. Compounds with higher activity were further screened for compounds with both high OB and DL, using OB > 30% and DL > 0.18 as thresholds (11). Target proteins corresponding to each component were then obtained in the TCMSP Database and converted to a unified gene name using the protein database UniProt^3^ (12).

### Identification of putative target genes for cancer-associated malnutrition

The GeneCards^4^ (13) and OMIM^5^ (14) databases were searched using the keyword “cancer malnutrition” and the species “Homo sapiens.” Retrieved gene data were merged and disease-related target data were obtained by filtering out duplicate values.

### Shiliao decoction and cancer-associated malnutrition target screening and network construction

A Venn diagram was built by mapping active ingredient targets to disease targets using the “Venn” function package in R software (15), and overlapping targets were identified as core targets of SLD for CAM therapy. A common target network was constructed using Cytoscape (version 3.8.2) (16).

### Construction of the protein-protein interaction network

Shared target data about drugs and diseases were imported into the STRING 11.0 database^6^ (17) to construct protein-protein interaction (PPI) networks. To ensure the robustness of the analysis, the screening threshold of the STRING database was set to an interaction score ≥ 0.9, and free proteins were removed. PPI networks were then visualized and analyzed using Cytoscape (16). To identify the central nodes and key proteins in the PPI network, topological parameters were calculated using NetworkAnalyzer. CytoNCA (18) was used to calculate degree, betweenness, and closeness in order to describe the topological importance of proteins in the network.

### Biological information: Gene ontology and genome encyclopedia pathway enrichment analysis

Target gene analysis was performed using the “cluster profile” and “pathview” function package in R, a well-known R package with dynamically updated data for KEGG and GO analysis. Results were screened using a threshold *p*-value of ≤ 0.05. Results were then visualized using the “ggplot2 package” in R software (version 4.2.0) to obtain a bubble map of the results of the GO and KEGG enrichment analyses. Biological process (BP), cellular component (CC), and molecular function (MF) are all included in the GO functional analysis.

### Prognostic values of hub genes and analysis of tumor-infiltrating immune cells

The Kaplan-Meier Plotter (19) is a powerful online tool for assessing the impact of 54,000 genes on survival in 21 different cancer types. We focused on the relationship between the expression of the height-degree value gene and survival, including overall survival (OS) and progression-free survival (PFS). Log-rank *P*-values with 95% confidence intervals (CIs) and hazard ratios (HRs) were calculated.

The TIMER database^7^ (20) contains 10,897 TCGA samples from 32 cancer types and is a suitable resource for the systematic study of immune infiltration in different cancer types. Key targets of SLD components were analyzed using the TIMER database to explore their relationship with immune cell infiltration (B cells, CD4^+^T cells, CD8^+^T cells, neutrophils, macrophages, and dendritic cells) and the association with prognosis.

### Differential expression analyses

Immunohistochemistry (IHC) can reveal the relative distribution and abundance of proteins by utilizing the high specificity of antibody-antigen binding. Immunohistochemical data were obtained from the Human Protein Atlas (HPA),^8^ and the expression of target genes in normal and cancer tissues was compared. Transcriptome data of cancer-related genes were obtained from The Cancer Genome Atlas (TCGA).^9^

### Construction and analysis of the component-target-pathway network

An appropriate component-target-pathway (C-T-P) network was constructed and visualized *via* Cytoscape (16) to analyze the association between SLD, candidate targets, and CAM-related pathways.

### Molecular docking analysis

2D structures of the 10 compounds with the highest degree of active ingredients were downloaded from the Pub Chem database^10^ (21) and saved in “SDF” format. Chem 3D was used to convert the “SDF” format into mol2 structures as small molecule ligands. The 3D structures of the top 6 proteins in terms of degree value were retrieved from the PDB database^11^ (22) and saved as protein receptors in “PDB” format. Water molecules were removed using PyMOL software (version 2.3.6), and the original ligands were isolated from core target proteins. The processed protein targets were imported into AutoDock software (version 4.2.0) (23) for hydrogenation, calculation of total charge, and setting of the atom type. Ligands and protein receptors were recorded in PDBQT format. Molecular docking was performed with AutoDock-Vina software (version 1.1.2) (24) to evaluate the affinity of the receptor-ligand complexes with a comprehensive score. Molecular docking was visualized using Discovery Studio and docking patterns were displayed in 2D and 3D structures. Docking effects were evaluated based on their affinity value, with affinity values < –5 kcal/mol interpreted as representing good binding interaction between compound and target (25).

## Results

### The main active ingredients of shiliao decoction and putative target genes for cancer-associated malnutrition

Target prediction of SLD ingredients using the TCMSP database and screening (for OB ≥ 30% and DL ≥ 0.18 as described in methods) identified 172 compounds with sufficiently high OB and DL. They consisted of 5 compounds from *Zanthoxylum bungeanum* Maxim., 4 compounds from *Zingiber officinale* Rosc., 5 compounds from *Citrus reticulata* Blanco., 6 compounds from *Typha angustifolia* L., 17 compounds from *Codonopsis pilosula* (Franch.) Nannf., 88 compounds from *Glycyrrhiza uralensis* Fisch., 6 compounds from *Coix lacryma-jobi L.var.ma-yuen* (Roman.) Stapf., 19 compounds from *Ziziphus jujuba* Mill., 9 compounds from *Amomum villosum* Lour., 12 compounds from *Dioscorea opposite* Thunb.

The UniProt database was used to search for information on potential targets of the active ingredients identified by the TCMSP database. Target gene names were standardized, and 261 target genes were obtained after removal of duplicates.

2,581 CAM-related genes were identified by searching the GeneCards database, and 168 CAM-related genes were separately identified in the OMIM database. Following combination of these two sets and removal of duplicates, a total of 2,706 CAM-related genes were selected for further analysis.

### Shiliao decoction and cancer-associated malnutrition target gene screening and network construction

In order to identify potential gene targets of SLD with an effect on CAM, the set of 2,706 CAM-related genes selected earlier was compared with the set of 261 target genes of SLD components. A total of 147 potential target genes were found to be members of both these sets.

**FIGURE 2:**
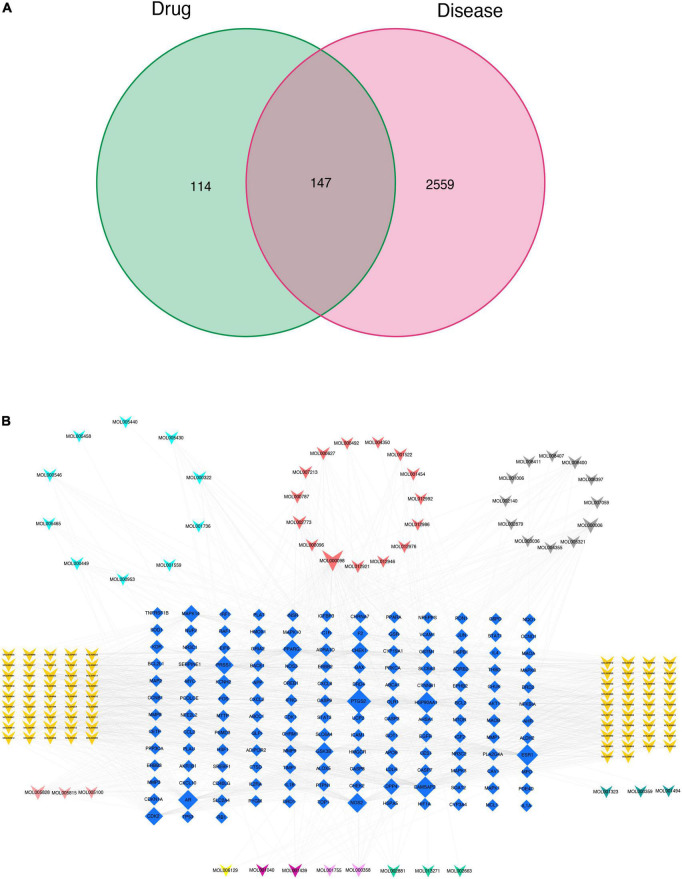
Construction of the target genes set **(A)** and compound-target network **(B)**. Blue quadrilateral nodes indicate targets, while differentially colored V nodes denote compounds. The dark yellow, light purple, light red, light blue, dark green, gray, dark red, dark purple, light yellow, and light green V nodes represent active compounds of *Glycyrrhiza uralensis* Fisch., *Amomum villosum* Lour., *Citrus reticulata* Blanco., *Dioscorea opposite* Thunb., *Coix lacryma-jobi L.var.ma-yuen* (Roman.) Stapf., *Codonopsis pilosula* (Franch.) Nannf., *Ziziphus jujuba* Mill., *Typha angustifolia* L., *Zingiber officinale* Rosc., *Zanthoxylum bungeanum* Maxim., respectively.

Cytoscape software was used to visualize the compound-target relationships and rank the components of SLD in descending order of degree. The top 12 key compounds are listed in Table 2, and the “compound-target” network consisting of 281 nodes and 1,360 edges, including 134 active components and 147 targets, is illustrated in Figure 2.

**TABLE 2:** Information table of key compounds of SLD.

Compound-ID	Compound name	Degree value
MOL000098	Quercetin	84
MOL000006	Luteolin	35
MOL000422	Kaempferol	32
MOL005828	Nobiletin	25
MOL004328	Naringenin	23
MOL003896	7-Methoxy-2-methyl isoflavone	22
MOL000392	Formononetin	21
MOL000497	Licochalconea	20
MOL000354	Isorhamnetin	20
MOL000500	Vestitol	17
MOL004957	HMO	17
MOL000358	Beta-sitosterol	17

### Protein-protein interactions network construction and key target screening

**FIGURE 3:**
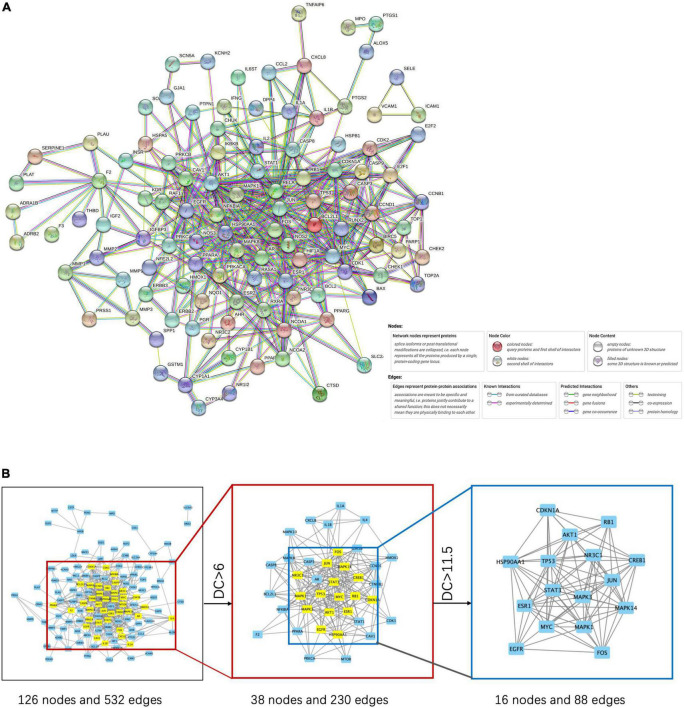
PPI network **(A)** and its key target network **(B)**.

We created a PPI network by importing the 147 target genes selected earlier into the STRING database. Cytoscape software was used to visualize the PPI network. Screening was carried out using a threshold score value of 0.9 to provide a high level of confidence for protein interactions, and unconnected nodes in the network were concealed, as seen in Figure 3A. Topological analysis was carried out using the CytoNCA plug-in. To identify core target genes, “intermediate centrality (BC), closeness centrality (CC), and degree centrality (DC) greater than the median” were utilized as screening criteria. This process is shown in Figure 3B, and specific information on the 10 core target genes is listed in Table 3.

**TABLE 3:** Topological parameters of the top 10 core targets.

Name	Betweenness	Closeness	Degree	Eigenvector	Network	Subgragh
JUN	180.374288	0.77083333	26	0.27641884	21.5991641	223861.828
TP53	88.6903298	0.7254902	23	0.2610248	17.064563	199612.516
MAPK3	91.2776653	0.7254902	23	0.26607129	17.7049667	207408.578
MAPK1	85.432815	0.71153846	22	0.25171047	16.1937549	185626.031
MAPK14	75.9562576	0.68518519	20	0.23496044	13.3996192	161742.578
STAT3	72.7535152	0.68518519	20	0.23960288	13.1754302	168192.469
AKT1	49.5316515	0.67272727	19	0.2281941	12.6352595	152559.438
HSP90AA1	53.8797507	0.63793103	18	0.20564286	12.145766	123908.617
FOS	51.7518378	0.64912281	17	0.20146626	10.3957445	118919.898
MYC	20.7274333	0.63793103	16	0.21363621	10.7609307	133712.031

### Gene ontology and genome encyclopedia enrichment analysis

**FIGURE 4:**
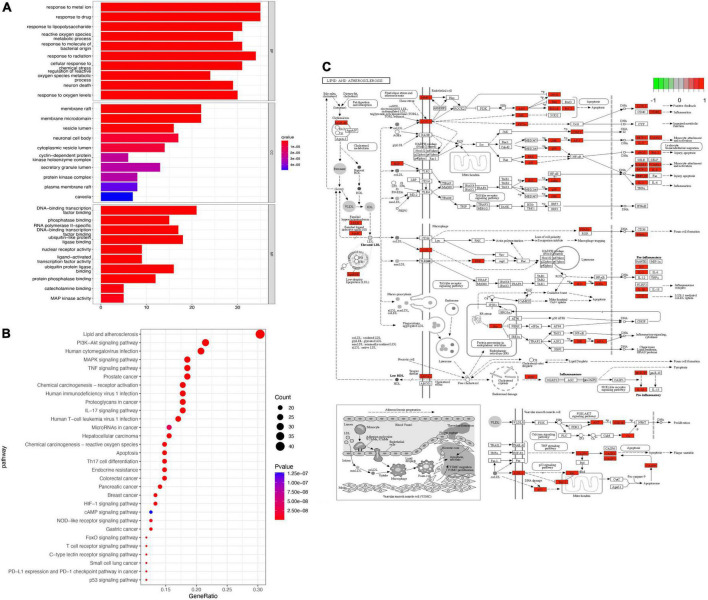
Enrichment analysis. **(A)** GO enrichment analysis. **(B)** KEGG enrichment analysis. **(C)** The most enriched KEGG pathway. Red genes in the network is the SLD target genes.

GO enrichment analysis found that BP mainly included pathways related to cellular metal ion response, response to drugs, and response to radiation. CC included the endocytotic activation pathway of membrane rafts and membrane microfilms among other pathways. MF mainly included pathways related to DNA binding transcription factor and ubiquitinated protein ligase (Figure 4A). Pathways identified by KEGG analysis included those involved in lipid and atherosclerosis, the PI3K-Akt signaling pathway, MAPK signaling pathway, TNF signaling pathway, cancer-related signaling pathways, and numerous immune-related signaling pathways (Figure 4B). Among these enriched pathways, we found that lipid and atherosclerosis signaling pathways play an important role in CAM (Figure 4C).

### Prognostic values and immune infiltration

**FIGURE 5:**
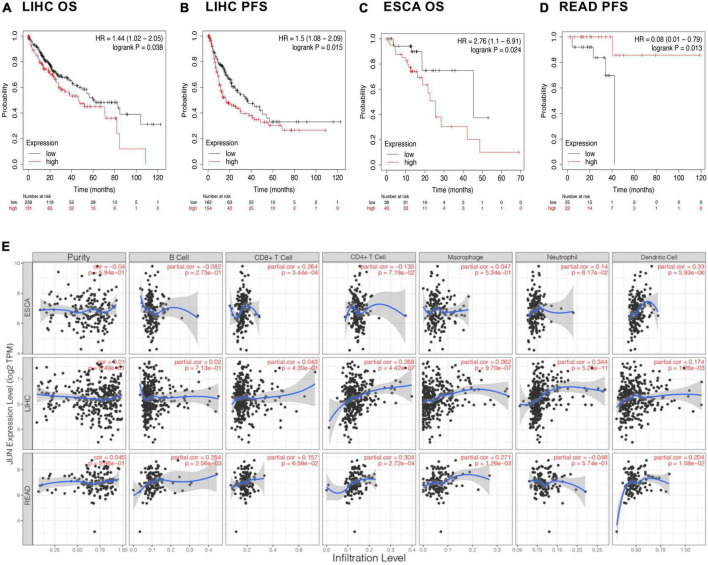
Immune infiltration and prognosis. **(A–D)** Association of *JUN* with the prognosis of different cancers. **(E)** Correlation of *JUN* expression with immune cells.

Based on these results, we selected *JUN* as a representative gene and focused on cancer types with co-occurring malnutrition to explore the association between them. Significant correlations (*P* ≤ 0.05) were found between *JUN* expression levels and survival in patients with liver hepatocellular carcinoma (LIHC), esophageal squamous carcinoma (ESCA) and rectal adenocarcinoma (READ), as shown in Figures 5A–D.

To further explore possible immunological mechanisms, we looked at the relationship between gene expression, tumor tissue purity, and the abundance of immune infiltrating cells in the microenvironment using the TIMER database. Tumor tissue purity is the proportion of cancer cells in a tumor sample, which is critical to selecting genes related to deconvolving immune cells in the tumor tissue. This was inferred from copy number alteration data using the R package CHAT (26). As seen in Figure 5E, it was observed that *JUN* expression levels did not significantly correlate with tumor purity, but more likely affected prognosis by regulating the tumor microenvironment. For example, in ESCA, *JUN* expression was positively correlated with infiltration of CD8^+^ T cells and dendritic cells. In LIHC, *JUN* expression was positively correlated with infiltration of CD4^+^ T cells, macrophages, and neutrophils while in READ, *JUN* expression was positively correlated with CD4^+^ T cell, macrophage and dendritic cell infiltration.

### Differential expression of JUN in cancers

**FIGURE 6:**
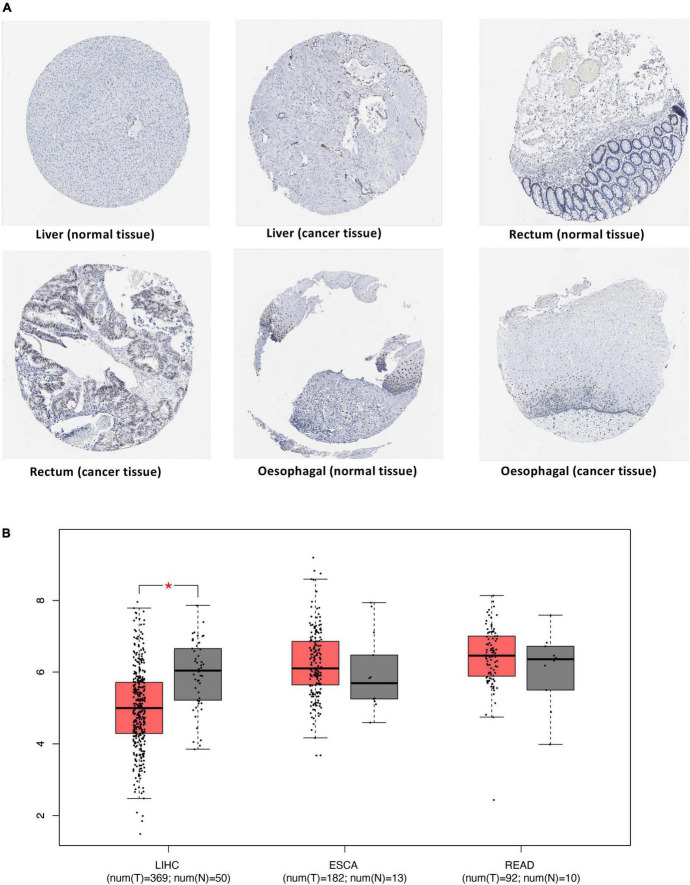
Differential expression of JUN in cancers. **(A)** Validation of the expression of JUN at the protein level using the HPA database. **(B)** Validation of the expression of *JUN* at the transcriptome level using the TCGA database.

To verify the potential of the identified proteins as therapeutic targets, we analyzed the differential expression of proteins in cancer and cancer-adjacent tissues. IHC results from the HPA database showed that JUN expression in cancer tissues was higher than that in normal liver and rectum tissues. On the other hand, antibody staining levels of JUN in ESCA cancer tissues were lower than those in adjacent tissues (Figure 6A).

The TCGA database was used to expand the sample size for validation and obtain expression data at the transcriptome level for this gene. The box plot of differential expression analysis (Figure 6B) showed that *JUN* was significantly overexpressed in normal tissues than in liver cancer tissues.

### Component-target-pathway network and molecular docking

**FIGURE 7:**
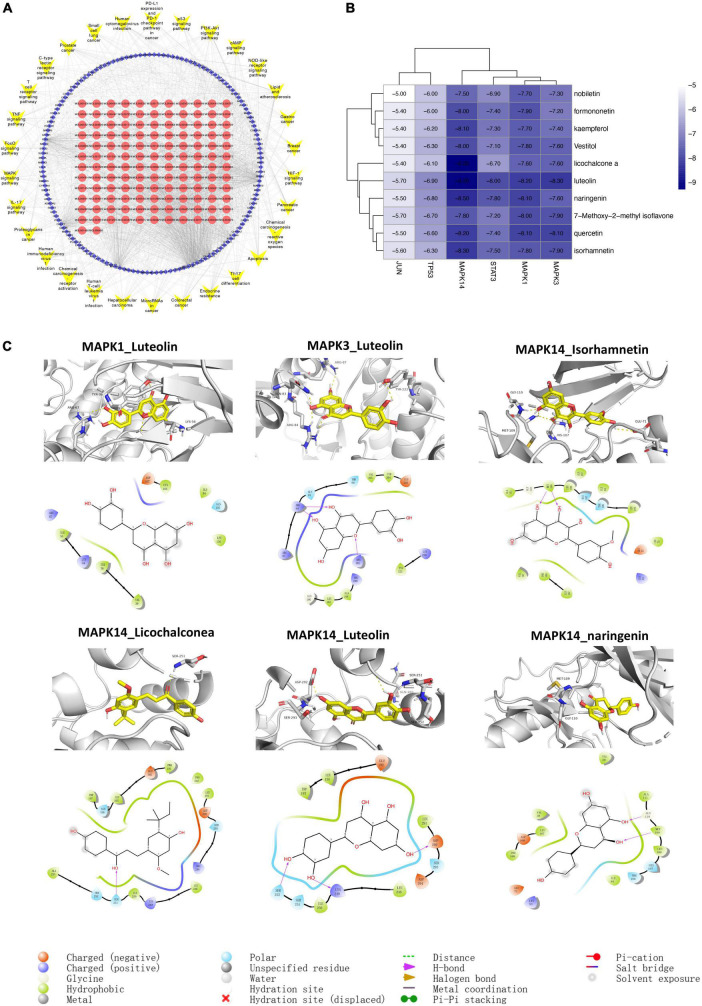
Component-target-pathway network **(A)** and molecular docking **(B,C)**. **(A)** The blue diamonds represent genes. The red circle nodes denote components of SLD, and the yellow V nodes represent CAM-related signaling pathways. **(B)** Molecular docking results of core targets and active compounds. The color indicates an affinity score. Dark blue represents the lowest affinity score and the highest affinity between receptor and ligand. White represents the highest affinity score and the lowest affinity between receptor and ligand. **(C)** Optimal complex structure of components and targets.

A “C-T-P” network was constructed based on the 30 most relevant signaling pathways obtained in Figure 4B. Quercetin, luteolin, kaempferol, nobiletin, naringenin, 7-Methoxy-2-methyl isoflavone, formononetin, licochalconea, and isorhamnetin were the elements with the most targets in this network, indicating that these substances may be the foundation of how SLD affects CAM. JUN (PDB ID:1A02), TP53 (PDB ID: 6wqx), MAPK3 (PDB ID: 6GES), MAPK1 (PDB ID: 7nr9), MAPK14 (PDB ID:6QE1), and STAT3 (PDB ID: 6tlc) were protein targets that connected with the most active components and pathways, suggesting that they may be key targets for treatment of CAM with SLD. It is likely that active components of SLD act through targets to jointly regulate signaling pathways involved in lipid and atherosclerosis, the PI3K-Akt signaling pathway, immune-related signaling pathways, and pathways in cancer to improve patient outcomes (Figure 7A).

We performed molecular docking between the top 6 protein targets in the “active ingredient-target-disease” network and the top 10 active ingredients in the SLD using Autodock vina. Based on similar experiments, binding energy < –4.25 kJ-mol-1 was interpreted as indicating that the active ingredient had some binding ability for the target, binding energy < –5.0 kJ/mol indicated good binding activity, and binding energy < –7.0 kJ/mol indicated strong binding activity (27). Analysis of molecular docking found that the binding energies of the above target proteins and compounds were less than –5 kcal/mol, indicating good binding activity. Detailed results are shown in Figures 7B,C.

## Discussion

Increased catabolism of fat, muscle, and other tissues is a clinical characteristic shared by malnutrition and cancer cachexia, a specific kind of CAM characterized by persistent inflammation (28). Notably, typical nutrition therapy does not completely reverse cancer cachexia. Refractory cachexia is an advanced stage of disease fundamentally arising from the combination of uncorrected nutritional impairment and lack of response to anticancer treatment. Nutritional issues of patients should be thought of as existing along a continuum, stretching from the first signs and symptoms of anorexia to precachexia, cachexia, and refractory cachexia (29). It is well known that the efficacy and the impact of nutritional interventions are linked to the timing of such support, with the best results obtained with early intervention. However, there are no effective medical interventions in Western medicine that can completely reverse the conditions of CAM and cachexia. By contrast, TCM containing phytonutrients has good efficacy and safety and should be explored in greater depth.

The TCM expert and one of the pioneers of Chinese medical oncology Professor Zhou Daihan proposed that Chinese medical oncology food therapy is a discipline based on the theory of the doctrine of TCM organs and meridians, combined with modern oncology and nutrition to guide people to cure tumors through dietary changes (30). According to the latest document “National Health Food Convenience Letter (2018) No. 8” issued by the National Health Commission of China in 2018, 110 Chinese botanical drugs are recognized as medicinal and food ingredients and promoted for appropriate medical use (31). Based on the basic system theory of Chinese medicine, our team selected ten botanical drugs to make up SLD. Through network pharmacological research, the primary active components of SLD for CAM treatment were identified as quercetin, luteolin, kaempferol, nobiletin, naringenin, 7-Methoxy-2-methyl isoflavone, formononetin, licochalconea, and isorhamnetin. Quercetin, luteolin, kaempferol, nobiletin, naringenin and isorhamnetin are all flavonoids, which possess strong anti-inflammatory and anti-tumor effects both *in vitro* and *in vivo*. Flavonoids have been shown to lower triglyceride and cholesterol levels and reduce inflammatory mediators (32), as well as activate the monocyte/macrophage pathway. This suggests that flavonoids may contribute to treatment of CAM by regulating inflammation, immunity and lipid metabolism (33). For example, luteolin has been found to reduce cancer-induced skeletal muscle atrophy in a Lewis lung cancer mouse model by a mechanism that inhibits TNF-α and IL-6 directly and can suppress inflammation levels to treat cachexia by reducing NF-κB activation at the transcriptional and translational level (34). A 2021 study using mice with C26 cells found that naringenin prevented the loss of muscle strength and decreased levels of insulin resistance and inflammation (35). Many reports have demonstrated that kaempferol is a safe and effective natural dietary anti-inflammatory agent, and the poor bioavailability of kaempferol has also been addressed by nanotechnology (36). Kaempferol has been shown to protect against chemotherapeutic drug-induced cardiotoxicity and improve advanced tumor cardiac cachexia by inhibiting p53-mediated mitochondria-dependent apoptotic signaling and regulating in ERK-dependent mitogen-activated protein kinase pathway in *in vitro* and *in vivo* assays (37).

In this study, JUN, TP53, MAPK3, MAPK1, MAPK14, STAT3, AKT1, HSP90AA1, FOS, and MYC were finally selected based on the PPI network and major targets of the C-T-P network map. Molecular docking studies have shown that SLD has a good affinity for the products of many of these gene targets. JUN, a prominent member of the activator protein 1 (AP-1) family, plays an important role in processes including cell proliferation, differentiation, and tumor transformation (38). A study using an animal model of prolonged fasting malnutrition showed that metabolic control in metabolically active organs is exerted by transcription factors, including JUN, activated by nutritional signaling (39). Consistent with this, the presence of anti-c-Jun antibodies has been shown to reduce the nuclear activator protein AP-1 binding activity in protein energy-deficient dystrophic rats (40). A clinical study also found oxidative modifications and ubiquitination of Jun-D in skeletal muscle of patients with cancer cachexia, suggesting that this factor is also associated with cachectic muscle atrophy (41). JUN is also a key target of c-Jun N-terminal kinase (JNK), a member of the mitogen-activated protein kinase superfamily (MAPK), which activates JNK, translocates it from the nucleus to the cytoplasm and phosphorylates Jun, thereby increasing its activation potential. Following cancer-induced activation of Toll-like receptor 4 in skeletal muscle, p38β MAPK phosphorylates Ser-12 on p300 to stimulate C/EBPβ acetylation. p38β MAPK has been shown to cause skeletal muscle atrophy by inducing autophagy in skeletal muscle cells (42). As such, it may be a central mediator and potential therapeutic target in cancer-induced muscle atrophy. Because nilotinib selectively inhibits p38β MAPK, systemic administration of nilotinib at low doses (0.5 mg/kg/day, ip) in tumor-bearing mice not only attenuates muscle atrophy but also prolongs survival (43). The cachexia phenotype is causally associated with the cytokine-activated transcription factor STAT3, which contributes to symptoms including skeletal muscle atrophy, cardiac dysfunction, and hypothalamic inflammation (44). Related studies have shown that STAT3 is associated with fat wasting and the acute phase response to cancer cachexia. STAT3 also contributes to cancer cachexia by enhancing tumorigenesis, metastasis and immunosuppression, particularly in tumor types associated with a high risk of cachexia (45).

GO gene enrichment and KEGG pathway analysis identified pathways affecting lipid metabolism and atherosclerosis, in addition to many immune-related pathways. Brown catabolism of fat is necessary for muscle catabolism in CAM, linking fat metabolism to CAM and indicating that it may be a prime target process for early therapy intervention (46). Animal studies have confirmed that quercetin inhibits cisplatin-induced fat loss by regulating the expression of genes involved in fat metabolism and synthesis as well as plasma TNF-α levels (47). Citrus flavonoids, including naringenin and nobiletin, can blunt the inflammatory response in metabolically important tissues including adipose tissue, and have emerged as promising therapeutic agents for the treatment of metabolic dysregulation (48). The above results suggest that SLD has the potential to intervene in CAM and curb the progression of cachexia at an early stage by reducing pathological inflammation and improving lipid metabolism. On the other hand, Luteolin activates the PI3K-Akt pathways in antigen presenting cells (APCs), induces the activation of APCs, enhances cytotoxic T lymphocyte responses, and inhibits tolerogenic T cells (49). An *in silico* docking simulation determines the detailed mode of binding of kaempferol to PD-1/PD-L1 and these results suggest that kaempferol can potentially be developed as a potent small molecule inhibitor for PD-1/PD-L1 blockade (50). Isorhamnetin has been identified as a negative regulator of pro-inflammatory cytokine release from CD4^+^ T lymphocytes and has also been shown to reduce the release of pro-inflammatory cytokines from primary human lymphocytes (51). Therefore, we hypothesize that SLD may improve the prognosis of tumor patients by influencing the immune microenvironment, a proposal that is supported by the K-M and TIMER databases.

However, this study had several limitations. First, since the data from online databases were based on evaluated, we may not have included undocumented or unverified chemicals or targets in our study. Second, the metabolic forms, effective components, and absorption mechanisms of bioactive components of SLD would ideally be studied in greater depth. Furthermore, it must also be noted that this decoction interacts with the gut microbiota following oral ingestion, and this process may be different in subjects with different cancers or microbiome populations. Future microbiota-based multi-omics studies in Chinese medicine are likely to provide richer insights (52).

## Conclusion

In conclusion, we identified luteolin, kaempferol, isorhamnetin, and several other compounds to be the main active components of SLD. Among their targets that may mediate anti-CAM effects are JUN, TP53, MAPK, STAT3, etc. The effect of SLD appears to be primarily mediated by control of the inflammatory response through lipid metabolism pathways, immune-related pathways, and reduction in tissue and organ depletion that translate into improved prognoses for patients with CAM. The results of our study provide recommendations for experimental validation and studies on clinical application.

## Data availability statement

The datasets presented in this study can be found in online repositories. The names of the repository/repositories and accession number(s) can be found in the article.

## Author contributions

SL and SJ wrote the manuscript, performed GO, KEGG. immune infiltration, survival analysis, expression differences, and molecular docking. HX modified the figures. PX edited and improved the manuscript. SL compiled the TCM database and target capture. SJ collated the disease target database. SZ directed the research and proposed changes to the manuscript. All authors reviewed the manuscript and approved the final version of the manuscript.

## Footnotes

1
http://mpns.kew.org

2
http://tcmspw.com/tcmsp

3
http://www.uniprot.org/uploadlists/

4
https://www.genecards.org/

5
https://www.omim.org/

6
https://string-db.org/

7
http://cistrome.org/TIMER/

8
https://www.proteinatlas.org/

9
https://portal.gdc.cancer.gov/

10
https://pubchem.ncbi.nlm.nih.gov/

11
https://www.rcsb.org/
